# Long-Term Influence of Paraspinal Muscle Quantity in Adolescent Idiopathic Scoliosis Following Deformity Correction by Posterior Approach

**DOI:** 10.3390/jcm10204790

**Published:** 2021-10-19

**Authors:** Hong Jin Kim, Jae Hyuk Yang, Dong-Gune Chang, Se-Il Suk, Seung Woo Suh, Yunjin Nam, Sang-Il Kim, Kwang-Sup Song

**Affiliations:** 1 Department of Orthopedic Surgery, Inje University Sanggye Paik Hospital, College of Medicine, Inje University, Seoul 01757, Korea; hongjin0925@naver.com (H.J.K.); seilsuk@unitel.co.kr (S.-I.S.); 2 Department of Orthopedic Surgery, Korea University Guro Hospital, College of Medicine, Korea University, Seoul 08308, Korea; kuspine@naver.com (J.H.Y.); spine@korea.ac.kr (S.W.S.); nam.yunjin@gmail.com (Y.N.); 3 Department of Orthopedic Surgery, College of Medicine, The Catholic University of Korea, Seoul 06591, Korea; sang1kim81@gmail.com; 4 Department of Orthopedic Surgery, Chung-Ang University Hospital, College of Medicine, Chung-Ang University, Seoul 06973, Korea; ksong70@cau.ac.kr

**Keywords:** adolescent idiopathic scoliosis, paraspinal muscles, cross-sectional area, posterior approach, computed tomography

## Abstract

Pedicle screw instrumentation (PSI) through posterior approach has been the mainstay of deformity correction for adolescent idiopathic scoliosis (AIS). However, changes in the quantity of paraspinal muscles after AIS surgery has remained largely unknown. The aim of this study was to investigate long-term follow-up changes in paraspinal muscle volume in AIS surgery via a posterior approach. Forty-two AIS patients who underwent deformity correction by posterior approach were analyzed through a longitudinal assessment of a cross-sectional area (CSA) in paraspinal muscles with a minimum five-year follow-up. The CSA were measured using axial computed tomography images at the level of the upper endplate L4 by manual tracing. The last follow-up CSA ratio of the psoas major muscle (124.5%) was significantly increased compared to the preoperative CSA ratio (122.0%) (*p* < 0.005). The last follow-up CSA ratio of the multifidus and erector spine muscles significantly decreased compared to the preoperative CSA ratio (all *p* < 0.005). The CSA ratio of the erector spine muscle was correlated with the CSA ratio of the psoas major (correlation coefficient = 0.546, *p* < 0.001). Therefore, minimizing the injury to the erector spine muscle is imperative to maintaining psoas major muscle development in AIS surgery by posterior approach.

## 1. Introduction

Adolescent idiopathic scoliosis (AIS) comprises three-dimensional deformities of the spine, including structural, lateral, and rotated curvature, with unknown etiology, presenting at or around puberty [1]. From a radiological view, surgical management in AIS is indicated when the Cobb’s angle >45° in the thoracolumbar or >50° in the thoracic curve preventing curve progression, achieving maximum permanent correction of the three-dimensional deformity, improve walking, in general functional aspects, and minimizing complications [1,2]. With the advent of the thoracic pedicle screw, pedicle screw instrumentation (PSI) has been widely applied to achieve three-dimensional correction and stable fixation in AIS [3].

Age-related structural change in paraspinal muscles has an important role for movement and stabilization of the spine [4,5]. Some studies suggest relationships between paraspinal muscles and global sagittal alignment in adult spinal deformity [6]. However, paraspinal muscle in AIS was observed in the asymmetric aspect in accordance with scoliotic curves. These curves showed a shortened muscle on the concave side and a lengthened muscle on the convex side of the curve [7,8]. Furthermore, asymmetric imbalances of the paraspinal muscles have been considered as contributing factors to scoliotic curve progression [9,10].

Although the impact on paraspinal muscle development in AIS has been studied in conservative treatment, there have been no reports of the long-term influence on paraspinal muscles in AIS following PSI with posterior approach [7,9]. Therefore, this study aimed to investigate long-term follow-up changes in paraspinal muscle volume for AIS following PSI with posterior approach.

## 2. Materials and Methods

This study was performed through a retrospective comparative analysis at a single institute where spinal deformity correction was performed routinely. The concept and procedures of the study were approved by our institutional review board. Informed consent was waived by the Institutional Review Board due to the retrospective design. The medical record data of 281 patients with AIS who underwent deformity correction using PSI by posterior approach were collected from 2002 to 2012. Among the 281 patients with AIS, the exclusion criteria of this study were (1) patients with non-idiopathic etiology (neuromuscular or congenital scoliosis), (2) patients with a history of revision surgery, (3) follow-up loss within 5 years, and (4) cases in which CT was not performed preoperatively or at last follow up. A total of 42 patients were included, and data from these patients were analyzed longitudinally.

All patients underwent posterior surgery using rod derotation (RD) and direct vertebral rotation (DVR) with PSI. Fusion levels were determined by Suk classification [3]. Pedicle screws were inserted segmentally on both sides of the lumbar curve, on the concave side, and in every other or every third vertebra on the convex side in the thoracic curve. A contoured rod to one-third exaggeration of the normal sagittal alignment was inserted into correction side and derotated 90° to transform scoliotic curve into thoracic kyphosis and/or lumbar lordosis. DVR was performed to correct rotational deformity after correcting the coronal and sagittal curves by RD [3]. All patients wore a thoracolumbosacral orthosis brace (TLSO) for three months after surgery without any specific rehabilitation.

All patient data were collected from the hospital database and retrospectively analyzed. Demographic and operative variables were gender, ages at surgery and at last follow-up, body mass index (BMI) at the time of surgery, Risser stage at the time of surgery, fused segments, thoracoplasty, and number of resected ribs. Radiological variables were coronal and sagittal spinopelvic parameters preoperatively, postoperatively, and at last follow up. Main curve and coronal balance were collected as coronal parameters and were measured by Cobb’s angle. Sagittal vertical axis (SVA), thoracic kyphosis (TK), and lumbar lordosis (LL) were collected as radiological parameters.

**Figure 1:**
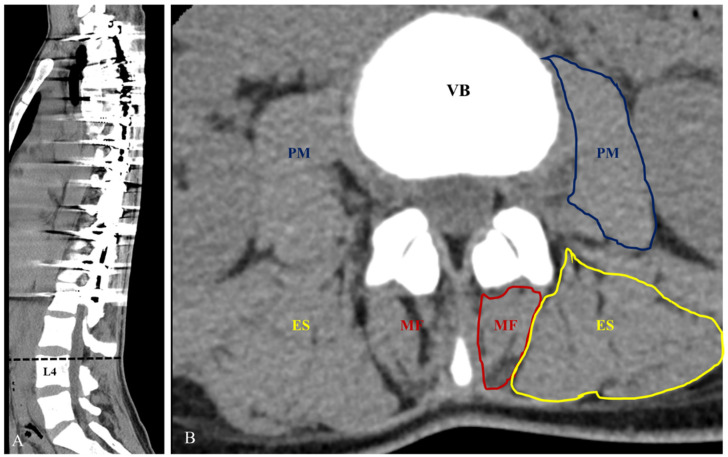
The cross-sectional areas (CSAs) of individual paraspinal muscles (multifidus (MF), erectus spine (ES), and psoas major (PM) muscles), and L4 vertebrae body (VB) were measured by assessment of axial computed tomography (CT) images. Measurements of the CSA of the paraspinal muscles were obtained at the level of the upper endplate of L4 by manual tracing (**A**,**B**).

The cross-sectional areas (CSAs) of individual paraspinal muscles (multifidus, erectus spine, and psoas major) and L4 vertebrae body (VB) were measured by assessment of axial CT images considering characteristics of motion segments and less affected vertebrae by deformity correction in included patients (Figure 1) [11]. The section from the upper endplate of the L4 vertebra was used. CSAs were measured bilaterally using the Picture Archiving and Communication Systems (PACS, INFINITT PACS, INFINITT Healthcare Company, Korea) to create a free line region of interest for each muscle. To minimize bias caused by individual relative body size and disk pathology, the CSA ratio was evaluated using the ratio of each muscle to VB (individual muscle CSA/L4 VB CSA), which was expressed as a percentage. The symmetry ratio of CSA between right and left paraspinal muscles was also evaluated. Measurement of the CSAs of the individual muscles and L4 vertebral body were carried out by two orthopedic surgeons to determine inter-examiner error. All parameters were measured 3 times with 2 week intervals to evaluate the intra-examiner reproducibility.

Statistical analysis was performed using SPSS Statistics for Windows, version 21.0 (IBM Corp., Armonk, NY, USA). A normal distribution was confirmed by the Kolmogorov–Smirnov test. Regarding continuous variables, a Student’s *t*-test (paired means) was used for parametric data. Longitudinal comparison of three groups used one-way repeated measures analysis of variance (ANOVA) and post hoc analysis used the Tukey honestly significant difference (HSD) test. Correlation of the CSA ratio with radiological parameters was analyzed using the Pearson correlation test. The intraclass coefficient (ICC) of individual CSA was measured to assess inter-examiner reliability with standardized agreement [12]. For variables having negative or positive values based on the measured reference point, such as coronal balance and SVA, statistical comparisons of groups required converting negative numbers to positive numbers because of the necessity to analyze differences from a reference point. Statistical significance was set at *p* < 0.05.

## 3. Results

### 3.1. Comparison of Patient Demographic Data

All demographic and operative data, including gender, age, body mass index (BMI), follow-up period, Risser stage at surgery, fused segments, thoracoplasty, and number of resected ribs are summarized in Table 1. A total of 42 patients (five males and 37 females) were enrolled in this study. The mean age at surgery and at last follow up was 14.6 years and 27.4 years, respectively, with a statistically significant difference (*p* < 0.001). The mean follow-up period was 9.9 years. For operative data, the mean number of fused segments was 11.1 and the mean number of resected ribs was 5.5 (Table 1).

The number of curve types by Suk’s classification as follows: nineteen (45.2%) single thoracic curves, 10 (23.8%) double thoracic curves, 10 (23.8%) double major curves, and three (7.2%) thoracolumbar/lumbar curves.

### 3.2. Comparison of Radiological Parameters

Regarding parameters of radiological outcomes, the mean correction rate was 72.7% and the loss of correction was 1.3°. The mean values of CB and SVA were within normal limits preoperatively, postoperatively, and last-follow-up. Last follow-up TK (32.2°) was significantly higher than preoperative TK (25.3°) (*p* < 0.001). For post hoc analysis, there was no statistical significance between preoperative TK and postoperative TK (*p* = 0.445). Last follow-up LL (44.6°) was significantly higher than preoperative LL (37.3°) (*p* < 0.001). With post hoc analysis, there was no statistical significance between preoperative LL and postoperative LL (*p* = 0.660) (Table 2).

### 3.3. Comparison of CSAs of the Paraspinal Muscles

**Figure 2:**
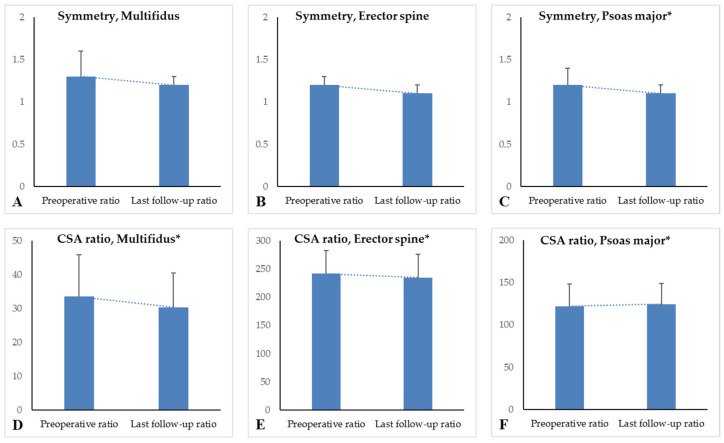
Symmetry difference between preoperative and last follow-up data. Symmetry was compared by ratio between large and small CSAs in the right and left paraspinal muscles. Only the symmetry of the psoas muscles showed statistical significance (**A**–**C**). The CSA ratio between preoperative and last follow-up data (**D**–**F**). Only the CSA ratio of the psoas muscle increased, from 122% to 124%, with statistical significance. * means statistical significance (*p* < 0.05).

The mean last follow-up CSAs of the multifidus, erector spine, and psoas major were significantly higher than the corresponding preoperative CSAs (all *p*-values < 0.001). Last follow-up CSA of the L4 vertebra body was 1179.9 mm^2^, which was significantly higher than the preoperative CSA of the L4 vertebra body (*p* < 0.001). The CSA ratio of the multifidus was 33.5% preoperatively and 30.4% at last follow up, a significant decrease (*p* = 0.001). The CSA ratio of the erector spine between preoperative and last follow up was 242.0% preoperatively and 235.0% at last follow up, a significant decrease (*p* < 0.001). Only the CSA ratio of the psoas major increased, from 122.0% to 124.5%, with statistical significance (*p* = 0.002) The symmetry ratio of multifidus was 1.3 preoperatively and 1.2 at last follow up (*p* = 0.209). The symmetry ratio of erector spine was 1.2 preoperatively and 1.1 at last follow up (*p* = 0.095). The symmetry ratio of psoas muscle was 1.2 preoperatively and 1.1 at last follow up, a significant improvement (*p* = 0.005) (Table 3) (Figure 2).

### 3.4. Correlation Analysis for CSA Ratios of Paraspinal Muscles

Analysis of correlation was performed between the last follow-up CSA ratios of paraspinal muscles and between the last follow-up CSA ratios of paraspinal muscles and radiological parameters. Correlations between last follow-up CSA ratio of psoas major and erector spine were significant with a correlation coefficient of 0.546 (*p* < 0.001). Correlations between last follow-up CSA ratio of psoas major and coronal balance were significant with a correlation coefficient of 0.314 (*p* = 0.043) (Table 4). 

All ICCs for the CSA ratio of multifidus, erector spine, and psoas major were greater than 0.75. Thus, all measurements of the CSA ratio showed excellent strength of agreement according to Fleiss guidelines (Table S1).

## 4. Discussion

Studies on the impact of paraspinal muscle quantity and quality on spinal diseases have been evaluated by CT and MRI [5,6,13]. The poor quantity and quality of paraspinal muscles resulted in degenerative lumbar kyphosis by quantitative analysis using CT [14,15,16]. However, there is no study of the changes in paraspinal muscles in the growth process after PSI by posterior approach in AIS. To the best of our knowledge, this is the first study investigating the long-term follow-up changes in paraspinal muscle volume in patients with AIS following deformity correction by posterior approach. Furthermore, our longitudinal study aimed to analyze the long-term follow-up changes in paraspinal muscles that are affected by injury during the growth process after deformity correction. AIS occurs more frequently in females and progress faster than in males. In our study, the enrolled patients were also mainly females.

Yeung et al. showed paraspinal muscle compositional change on the concave side by prolonged compression and reduced muscle activity in AIS, which illustrated that paraspinal muscle imbalances are associated with curve progression in AIS [10]. However, no studies have reported changes in paraspinal muscles after correction of scoliotic curve by surgery. In our study, correction rate and loss of correction showed no correlation with the CSA ratio of paraspinal muscles by Pearson’s correlation analysis. Therefore, even if paraspinal muscle imbalances affect scoliotic curve progression, the degree of correction does not significantly affect the development of paraspinal muscles after deformity correction by posterior approach. 

Even though PSI with posterior approach has been applied widely for AIS, surgery can lead to massive damage to the multifidus and erectus major muscles [2]. TK and LL were significantly increased to the normal range of an adult over the 9.9 year follow up of this study. From the post hoc analysis, last follow-up TK and LL significantly increased compared to preoperative values; this was thought to be the result of growth after deformity correction. Degeneration of the paraspinal muscles was related to sagittal imbalances in elderly groups [13,17]. Jun et al. illustrated that the quantity and quality of paraspinal muscles had greater influence on parameters of sagittal balance in elderly patients than in younger groups [6]. Our study showed that there were no correlations between paraspinal muscle and sagittal parameters in patients with AIS following deformity correction by posterior approach.

The psoas muscle of the deep back musculature plays a valuable role in the bolstering effect on the anterolateral aspect of the lumbar spine, stabilizing the spine in the upright position, and maintaining load-absorptive capacity [18]. Poor quantity and quality of the psoas muscle not only limit spinal movement, but also cause lower back pain [19]. In our study, the CSA ratios of paraspinal muscles significantly decreased during the 9.9 year follow-up period except for that of the psoas muscle. This was due to the fact of injury of the multifidus and erector spine muscles during deformity correction through posterior approach. The psoas muscle, as a deep muscle group, seems to have no significant restrictions on growth and development, because it is a less-damaged area during the deformity correction process. Furthermore, only the psoas muscle significantly improved for symmetry. It was also associated with deep muscle group, less-damaged area in deformity correction, and developed during growth process.

The role of the psoas muscle after PSI by posterior approach in AIS patients is important because of its effect on the load absorptive capacity. The psoas muscle strengthens adjacent spinal segments and reduces stress on the spinal fusion instrumentation segment [5,18,19]. For correlation analysis in our study, the CSA ratio of the psoas muscle correlated with that of the erector spine muscle. The degree of damage to the erector spine muscle could affect the development of the psoas muscle. Therefore, minimizing the injury to the erector spine muscle is imperative to maintaining psoas major muscle development after AIS surgery by posterior approach. Furthermore, last follow-up CB correlated with psoas muscle CSA ratio. In AIS patients, CB has a greater effect on the upright position than on sagittal parameters, which reflects the role of psoas muscle [9].

Minimal invasive scoliosis surgery (MISS) recently showed comparable radiological and clinical outcomes with fewer complications compared to conventional open scoliosis surgery [20,21]. The smaller incision in MISS has esthetic benefits in addition to preservation of paraspinal muscles [22,23]. Therefore, MISS could be an alternative procedure to COSS to preserve the paraspinal muscles. However, comparative studies of paraspinal muscle quantity between MISS and COSS are needed.

There were several limitations in our study. First, the number of patients was relatively small, and we utilized a retrospective design. Second, this study did not reflect the morphological quality of paraspinal muscles. Large multi-center comparative studies are needed to confirm our results. However, our study focused on paraspinal muscle quantity, which is associated with development of paraspinal muscles after deformity correction. Third, the evolution pattern of paraspinal muscle quantity could not be examined in this study because of radiation hazards by CT scan in especially young adolescents. Lastly, although various muscles can effect on growth process in AIS, this study only limited on paraspinal muscles by using spine CT. Further trials will be needed for influences of abdominal musculatures in AIS. Nonetheless, our study suggested that minimizing the injury to the erector spine muscle was important to maintain psoas major muscle development in AIS patients following deformity correction.

## 5. Conclusions

Minimizing the injury to the erector spine muscle is imperative to maintain psoas major muscle development in patients who underwent AIS surgery with posterior approach. A minimally invasive surgical technique with preservation of the erector spine muscle could be important for skeletally immature AIS patients.

## Figures and Tables

**Table 1:** Demographic and operative data of this study.

Variables	Cases (*n* = 42)	*p*-Value
Gender (*n* (%)) Male Female	5 (11.9%) 37 (88.1%)	- -
Age (years) At surgery At last follow up	14.6 ± 2.4 * 27.4 ± 3.6 *	<0.001
Follow-up period (years)	9.9 ± 2.4 *	-
BMI at surgery (kg/m^2^)	18.1 ± 3.1 *	-
Risser stage at surgery	3.1 ± 0.9 *	-
Fused segments	11.1 ± 2.4 *	-
Thoracoplasty (Yes:No)	40:2	-
Number of resected ribs	5.5 ± 1.7 *	-

* All values are expressed as mean ± standard deviation. Significant differences were accepted for *p* < 0.05. N = number; M = male; F = female; BMI = body mass index.

**Table 2:** Radiological parameters of this study.

Variables	Cases (*n* = 42)	*p*-Value
Coronal parameters		
Main curve Preoperative (°) Postoperative (°) Last follow up (°) Correction rate (%) Loss of correction (°)	59.9 ± 15.1 16.4 ± 11.5 15.7 ± 12.0 72.7 ± 12.6 1.3 ± 16.4	<0.001 * Pre vs. Post: <0.001 * Post vs. Last: 0.973 * Last vs. Pre: <0.001
Coronal balance (mm) Preoperative Postoperative Last follow up	13.4 ± 8.3 14.4 ± 10.9 8.3 ± 6.0	<0.001 * Pre vs. Post: 0.849 * Post vs. Last: 0.005 * Last vs. Pre: 0.024
Sagittal parameters		-
Sagittal vertical axis (mm) Preoperative Postoperative Last follow up	18.2 ± 17.5 22.1 ± 13.8 18.1 ± 12.1	0.319
Thoracic kyphosis (°) Preoperative Postoperative Last follow up	25.3 ± 10.2 27.4 ± 6.7 32.2 ± 8.7	<0.001 * Pre vs. Post: 0.536 * Post vs. Last: 0.002 * Pre vs. Last: 0.001
Lumbar lordosis (°) Preoperative Postoperative Last follow up	37.3 ± 11.4 39.5 ± 10.2 44.6 ± 12.6	<0.001 * Pre vs. Post: 0.660 * Post vs. Last: 0.032 * Last vs. Pre: 0.012

Data are presented as mean ± standard deviation values for each group. *p*-Values are calculated by one-way repeated measures ANOVA test. * Post hoc analysis was performed by the Tukey HSD test. Significant differences were accepted for *p* < 0.05. N = number; Pre = preoperative; Post = postoperative; Last = last follow up.

**Table 3:** Longitudinal comparison of paraspinal muscle cross-sectional areas between preoperative and last follow-up data.

Variables	Preoperative (*n* = 42)	Last Follow Up (*n* = 42)	*p*-Value
CSA (mm^2^)			
Multifidus (mm^2^) Right Left Mean	173.9 ± 54.5 170.9 ± 76.1 172.4 ± 59.3	185.5 ± 61.1 170.4 ± 60.6 177.9 ± 57.4	0.001 0.015 <0.001
Erector spine (mm^2^) Right Left Mean	1291.3 ± 280.6 1229.1 ± 250.2 1260.2 ± 243.1	1478.5 ± 343.1 1261.5 ± 272.4 1370.0 ± 291.8	0.001 0.002 <0.001
Psoas major (mm^2^) Right Left Mean	609.3 ± 168.1 656.4 ± 145.7 632.8 ± 144.9	737.4 ± 197.2 728.3 ± 201.2 732.8 ± 193.3	<0.001 0.001 <0.001
L4 vertebrae body (mm^2^)	1046.3 ± 132.9	1179.9 ± 183.0	<0.001
Symmetry of CSA			
Multifidus Erector spine Psoas major	1.3 ± 0.3 1.2 ± 0.1 1.2 ± 0.2	1.2 ± 0.1 1.1 ± 0.1 1.1 ± 0.1	0.209 0.095 0.005
CSA ratio (%)			
Multifidus Erector spine Psoas major	33.5 ± 12.4 242.0 ± 40.6 122.0 ± 26.6	30.4 ± 10.1 235.0 ± 41.0 124.5 ± 27.4	0.001 <0.001 0.002

Data are presented as mean ± standard deviation values for each group. *p*-Values were calculated by paired *t*-tests. Significant differences were accepted for *p* < 0.05. Mean CSAs of paraspinal muscles were calculated as the average of right and left CSAs of paraspinal muscles. CSA ratios were evaluated as each muscle CSA to that of the L4 vertebra body expressed as a percentage. N = number; CSA = cross-sectional area.

**Table 4:** Pearson’s correlation analysis between last-follow-up paraspinal muscle and the measured parameters.

Parameters of CSA Ratio	Comparative Parameters	Correlation Coefficient	*p*-Value
Multifidus	Age Duration BMI Correction rate Loss of correction CB (Last follow up) SVA (Last follow up) TK (Last follow up) LL (Last follow up)	0.048 0.193 0.146 −0.266 0.177 0.023 0.046 0.239 0.081	0.765 0.221 0.355 0.088 0.262 0.886 0.770 0.127 0.611
Erector spine	Age Duration BMI Correction rate Loss of correction CB (Last follow up) SVA (Last follow up) TK (Last follow up) LL (Last follow up) Multifidus CSA ratio	0.304 0.179 −0.280 0.202 0.041 0.160 −0.197 0.281 0.294 0.039	0.051 0.257 0.073 0.200 0.795 0.312 0.211 0.072 0.058 0.808
Psoas major	Age Duration BMI Correction rate Loss of correction CB (Last follow up) SVA (Last follow up) TK (Last follow up) LL (Last follow up) Multifidus CSA ratio Erector spine CSA ratio	0.095 0.089 −0.187 0.268 0.029 0.314 −0.127 0.027 0.035 −0.116 0.546	0.549 0.575 0.235 0.087 0.855 0.043 0.423 0.865 0.827 0.465 <0.001

Significant differences were accepted for *p* < 0.05. CSA = cross-sectional area; BMI = body mass index; CB = coronal balance; SVA = sagittal vertical axis; TK = thoracic kyphosis; LL = lumbar lordosis.

## Data Availability

Data collected for this study, including individual patient data, will not be made available.

## Supplementary Materials

The following are available online at https://www.mdpi.com/article/10.3390/jcm10204790/s1, Table S1: Intra-examiner reproducibility and inter-examiner reliability by intraclass correlation coefficient.
