# Supplementary material for: Long-Term Influence of Paraspinal Muscle Quantity in Adolescent Idiopathic Scoliosis Following Deformity Correction by Posterior Approach
Source: J Clin Med. 2021 Oct 19;10(20):4790. doi: 10.3390/jcm10204790 (PMC8538741; doi:10.3390/jcm10204790)
Supplement: Supplementary file 1 [file jcm-10-04790-s001.zip › jcm-1347208-supplementary.pdf]

Table S1. Intra-examiner reproducibility and inter-examiner reliability by intraclass correlation coefficient

Variables	Intra-Examiner		Inter-Examiner	
CSA ratio	ICC	Strength of Agreement	ICC	Strength of Agreement
Multifidus (%)	0.80	Excellent	0.76	Excellent
Erector spine (%)	0.91	Excellent	0.85	Excellent
Psoas major (%)	0.84	Excellent	0.79	Excellent

Inter-rater reliability was interpreted with the Fleiss guidelines. According to Fleiss guidelines, strength of agreement was set as follows: ICC < 0.4: poor, ICC: 0.4 to 0.75: fair to good, and ICC: 0.75 to 1.00 excellent. ICC = intraclass correlation coefficient.
